# Hybrid social learning in human-algorithm cultural transmission

**DOI:** 10.1098/rsta.2020.0426

**Published:** 2022-07-11

**Authors:** L. Brinkmann, D. Gezerli, K. V. Kleist, T. F. Müller, I. Rahwan, N. Pescetelli

**Affiliations:** ^1^ Center for Humans and Machines, Max Planck Institute for Human Development, Lentzeallee 94, Berlin 14195, Germany; ^2^ Department of Humanities and Social Sciences, New Jersey Institute of Technology, Newark, NJ, USA

**Keywords:** cultural evolution, human–machine collaboration, social learning, transmission chain

## Abstract

Humans are impressive social learners. Researchers of cultural evolution have studied the many biases shaping cultural transmission by selecting who we copy from and what we copy. One hypothesis is that with the advent of superhuman algorithms a hybrid type of cultural transmission, namely from algorithms to humans, may have long-lasting effects on human culture. We suggest that algorithms might show (either by learning or by design) different behaviours, biases and problem-solving abilities than their human counterparts. In turn, algorithmic-human hybrid problem solving could foster better decisions in environments where diversity in problem-solving strategies is beneficial. This study asks whether algorithms with complementary biases to humans can boost performance in a carefully controlled planning task, and whether humans further transmit algorithmic behaviours to other humans. We conducted a large behavioural study and an agent-based simulation to test the performance of transmission chains with human and algorithmic players. We show that the algorithm boosts the performance of immediately following participants but this gain is quickly lost for participants further down the chain. Our findings suggest that algorithms can improve performance, but human bias may hinder algorithmic solutions from being preserved.

This article is part of the theme issue ‘Emergent phenomena in complex physical and socio-technical systems: from cells to societies’.

## 1. Introduction

When the first superhuman computer program in the game of Go—AlphaGo—beat the world champion Lee Sedol in 2016, its gameplay was considered surprising and unconventional, apparently violating longstanding Go traditions. In particular, for move 37, AlphaGo calculated the chance of a human player making the same move as 1 in 10 000 [1]. Its unconventional play likely originated from the fact that AlphaGo, and more so its successor AlphaGo Zero [2,3], learned through self-play with little or no reliance on human historic gameplay. The performance of AlphaGo raises the question of how such novel gameplay would influence human strategies [1]. Replaying historic human matches of the last 300 years showed that an algorithm similar to AlphaGo Zero increasingly often chooses the same move as humans [4], indicating convergence towards a common gameplay. Remarkably, there has been a steep increase in this alignment since 2017 when such an algorithm became available to the public [4,5]. These observations suggest the fascinating hypothesis that increased alignment is the result of a hybrid form of social learning, where AI solutions are copied and maintained by humans. Similar patterns of increased alignment between human and algorithmic play have been suggested in the game of chess [6].

The use of technology, such as books or software, for human training in games like Go and chess is not a novel phenomenon and represents a common method of socially transmitting knowledge from one generation to the next. Yet, current development in AI has made it possible for algorithms to not only play chess, but to play creatively without the need to rely on human games. This opened up the possibility of social learning—namely learning by observation [7]—between artificial and biological agents. Digital technology already influences the processes of social transmission among people by providing new and faster means of communication and imitation [8,9]. Going one step further, we argue that rather than a mere means of cultural transmission (such as books or the Internet), algorithmic agents and AI may also play an active role in shaping cultural evolution processes online where humans and algorithms routinely interact.

The influence of algorithms on human culture is increasingly coming under investigation. Much work has focused on the influence on cultural consumption by recommendation engines that create personalized rankings of, for instance, video clips or news [10,11]. On the production side of culture, algorithms are likewise gaining traction. For instance, in engineering or professional gaming, algorithms are involved in the design of products or provide new strategies. If and under which circumstances such algorithmic solutions merge with the human cultural repertoire remains an open question. In this study, we investigate social learning and reproduction of algorithmic behaviour, which might be a precondition for persistence within human culture.

We propose and test the hypothesis that social learning between humans and algorithms may be especially beneficial when biological and artificial problem solvers show diversity in the heuristics and strategies they adopt to problem-solving. Diversity in information, biases and problem-solving strategies has been suggested to reduce herding and error cascades [12–15]. By self-learning or by design, algorithms showing complementary biases to humans could foster the discovery of new solutions in domain-specific problems and improve outcomes compared to human-only problem solvers. We would expect this effect to be greater in domains where human bias is suboptimal for the problem to be solved. Algorithms learning from interaction with their environment, rather than from human data, may be able to innovate over human solutions, as in our opening anecdote. Similarly, when human biases are known, algorithms can intentionally be designed to exhibit complementary biases to their human counterpart to enhance collective performance [16]. Although several heuristics that humans employ are adaptive under assumptions of cognitive constraints and bounded rationality [17–19], they can be suboptimal under restricted circumstances—e.g. in digital environments to which they are not adapted [20–23]. In this study, we focus on a specific human bias, namely the tendency for myopic behaviour when facing a sequential decision [24–26].

**Figure 1.**
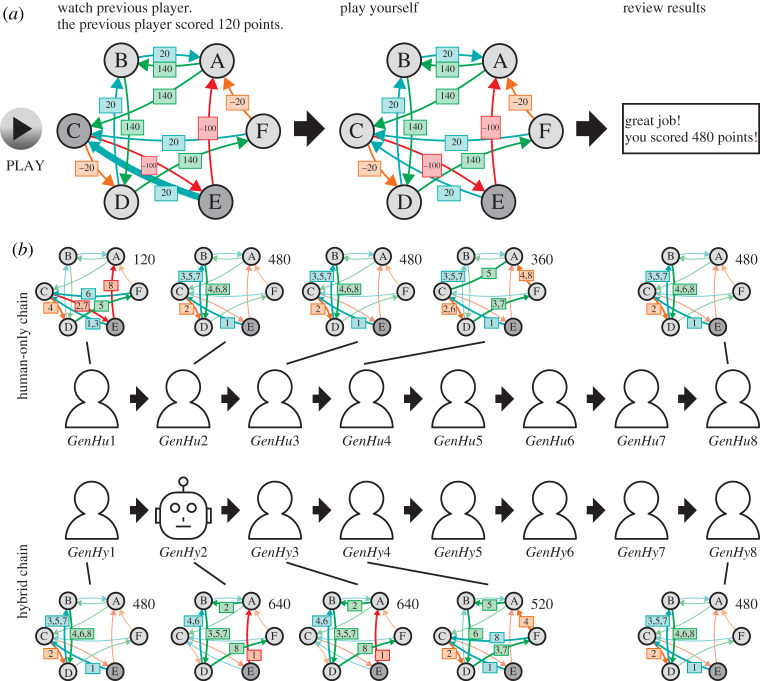
(*a*) In the first stage of the task, participants saw an animation of the solution entered by the previous player (left-hand side). A snapshot showing the transition from node E to node C is depicted. In the second stage, the participants could enter a path by clicking on the respective nodes in sequence (centre). The node with grey background colour indicates the current node the participant is in. In the last stage, the total score of the player’s sequence is revealed (right-hand side). The network presented here is classified as human-regretful. (*b*) For each environment class, we constructed two chains of eight generations of players. In hybrid chains, the second generation player was replaced by an algorithm. The networks depict the solutions of the first four generations as well as the last generation for a selected environment (corresponding to (*a*)). The integer on the arrows denotes the step at which a player was choosing the move. The cumulative reward is shown in the upper right corner of each graphic. In this example, for the human-only chain the cumulative reward increases at first, but quickly reaches a plateau. For the hybrid chain, the algorithm shows a performance greater than observed in the human-only chain, but this improvement gets lost over subsequent human generations. (Online version in colour.)

Many decision-making tasks (including Go and chess) are composed of sequential decisions that require an agent to explore large decision trees. As the tree grows exponentially large with increasing number of decisions, humans and algorithms rely on various heuristics to avoid exploring the full tree [3,26,27]. Huys *et al.* [26] introduced a goal-directed decision-making task where participants plan and make sequential moves on a directed network (figure 1). Each move is associated with gains or losses of different magnitude. Participants have full knowledge of the network and the rewards associated with each transition between two nodes. The authors found that people tend to selectively discount decision branches following a large cost [26,28]. We call this selective discounting aversive pruning bias. Such a heuristic can lead to sub-optimal solutions when an initial cost has to be born before a larger reward can be gained [29]. Lieder *et al.* showed that an algorithm can augment human decisions by providing pseudo-rewards (e.g. gamified badges and direct rewards) that reshapes the value of each option based on an optimal value function so as to render people’s myopic strategy optimal [29]. They showed that such pseudo-rewards can significantly increase participants' performance. Similar asocial algorithmic aids to human cognition have been investigated [30], but it remains unclear whether the associated benefits disappear when the aid is removed. We address the question of whether algorithms can durably improve human performance via social learning and whether humans further transmit such new behaviour to new human imitators. Social learning can be especially useful in complex problems and under uncertainty [7,31–33], and it does not require explicit causal understanding to be effective [34].

This paper explores hybrid social learning in the lab, adapting Huys’s decision-making task to a transmission chain paradigm with human and algorithmic players [35–37]. In a transmission chain, players solve a task in a sequence and can observe (and copy) the solution of the previous player before they enter theirs [38]. We compare a control condition of human-only players with a hybrid treatment condition where an algorithm replaced a human player in the second generation. Transmission chains have been used to investigate how biases in social learning shape cultural evolution [38–40]. Previous research has shown that humans have different biases of what (content bias) and who (context bias) is copied [7,41]. Both content and context biases are likely to be important in hybrid social learning. For instance, people differ in how they develop and sustain trust in human and algorithmic partners [42,43]. As we were interested in what people learn from artificial players, we controlled context bias by not revealing whether the previous player in the transmission chain was a human or an algorithm.

Participants repeatedly playing on the same network tend to reuse similar actions [28]. To exclude such asocial learning, we developed a novel randomized version of the task, in which each participant plays the same network only once. We classified environments where the human aversive pruning bias is adaptive (human-rewarding environments) or misadaptive (human-regretful environments). An agent with aversive pruning bias would perform well in the former and poorly in the latter. We designed the algorithmic player to show a bias opposite to humans, namely a tendency to explore decision branches associated with initial costs. We predicted performance improvement over generations and better performance in hybrid chains than in human-only chains (control) due to the increased strategic diversity of the former. In line with our preregistered hypotheses, we found increased performance over generations and a short-term performance improvement in the generation after the algorithmic solutions was introduced. However, in contrast to our initial hypothesis, the improvement introduced by the algorithm was not sustained over following generations of players. Solutions that conflict with the human aversive pruning bias had lower copying fidelity and therefore quickly disappeared. We develop an agent-based model that replicates some of our findings and makes novel predictions about untested experimental conditions. We discuss our results in terms of content bias and frequency of encounters with algorithmic solutions.

## 2. Methods

### (a) Participants

All 177 participants were recruited through Prolific (www.prolific.co), where they were redirected to an external website to complete the experiment. Before starting, they completed a consent form and read the instructions. The experiment, including two practice rounds, took around 60 min in total. Participants were paid £7 for the completion of the experiment. Furthermore, there was a reward of one penny given for every 100 points gained during the experiment. Participants received on average £3.20 bonus payments, depending on their performance. In cases where participants had to drop out because of technical issues (failed network connection, etc.), they were paid a compensation of £3.50. The experiment was run in multiple sessions. Two sessions failed for technical reasons. Data from failed sessions were disregarded entirely and the experiment restarted from the last saved image. The only entry requirement was speaking English. All participants were included in the analyses.

### (b) Task

The task was an adaptation of Huys *et al.* [26], in which participants were asked to find an optimal sequence of moves on a carefully designed directed network of six nodes. We generalized the task, by randomly sampling networks, instead of using a single network. From each node, there were exactly two possible moves to other nodes, each being associated with one of four possible payoffs (−100, −20, 20 or 140) (figure 1*a*). The full network, including all possible moves and their payoffs, was visible to the participant. Possible moves were visualized by directed arrows with colours coding for their respective payoffs (red, orange, blue and green for increasingly larger payoffs). The aim was to find a path of eight moves which maximizes cumulative payoffs, beginning at a fixed starting node. We called a network together with a specific starting position an environment. The experiment was implemented using a customized version of the Empirica framework [44] and consisted of three consecutive stages (figure 1*a*). In the first stage, participants were asked to watch the moves of the previous player's attempt to find the optimal solution. They saw the score of the previous player and a 15 s animation of the eight moves. The moves were animated on the same environment the participant played on. All moves were animated sequentially for about 2 s each with the start and target nodes being highlighted by a darker colour and the corresponding directed arrow and reward thickened (electronic supplementary material, figure S1 and video S1). In the second stage, participants were then asked to select a path of eight moves. The path could be entered by clicking on the nodes in sequence. The currently occupied node was displayed in a darker colour. If a node was selected which could not directly be reached from the current node, the erroneously selected node was coloured in bright red. The participant was then able to select a different node instead. Participants were able to see their current accumulated score, the number of steps remaining and a score of the last moves entered. This information was immediately updated whenever a participant clicked a possible target node (electronic supplementary material, figure S2). The answer of the participant was considered to be valid if all eight moves were played in the allotted time (15 s). Of all solutions entered by human participants, 9% were invalid. As invalid solutions were not considered in the formation of the chains, those were also omitted from the analysis. To strongly incentivise participants to respond even if they were uncertain about the solution, participants paid a large cost (−500) for the round if they did not provide a valid answer on time. In the third and final stage, the final score of the current round was displayed for 5 s in large fonts (electronic supplementary material, figure S3). Additionally, participants were informed if they had failed to enter a response on time.

#### (i) Experimental design

Transmission chains featured eight different players, who could be human or algorithmic. We call the position in the chain a generation. Within each chain, each player was exposed to the solution of the previous player. Players in the first generation were exposed to a random solution.

We manipulated the chain type (human versus hybrid, figure 1*b*) and the environment type (human-rewarding versus human-regretful), in a 2×2 design. In human-only chains (control condition), all eight generations featured human players. In hybrid chains (treatment condition), an algorithm (described below) replaced a participant in the second generation and provided an algorithmic solution instead. The rest of the chain comprised human players. 800 environments of two different types were investigated. The two types, ‘human-rewarding’ and ‘human-regretful’, differed in whether aversive pruning respectively increases or reduces the expected reward (see below for further detail). For each of the 800 environments two chains where constructed, one for each of the two chain conditions. This led to a total of 1600 chains and 12 800 games, of which 800 were played by the algorithm.

Participants were assigned to new environments on the fly at random based on availability, with the constraints that (a) the previous generation in the chain had successfully completed all stages and (b) that participants played each environment at most once. If a participant did not enter a path of eight moves on time, the solution was considered invalid and the corresponding position in the chain was reopened for a new participant. Each participant played a maximum of 80 rounds. Towards the end of the experiment, participants completed less than 80 rounds as no further environments were available. Due to the random assignment procedure, participants were equally likely to play in each of the chain types as well as the environment types throughout the experiment. However, participants entering the experiment at the beginning were more likely to be placed in earlier generations, compared to participants who entered the game at a later stage. Hence, we added to our regression models a random effect for each individual participant and, to control for individual experience with the task, we added a fixed effect for the number of rounds already played in the experiment.

#### (ii) Aversive pruning model

Huys *et al.* described a pruned tree search algorithm for this type of task that best fitted human decisions [26]. The model calculated the state-action value Q(a,s) of each action (move) a in state s. The value of a particular action is given by the sum of the immediate reward R(a,s) and the maximum value of the next action $a^{\prime }$ from the next state $s^{\prime }=T(a,s)$ where T is the deterministic transition function. At each level of depth of the search tree, future rewards are discounted by a factor of $\left(1-\gamma_{a,s}\right)$. Together, this leads to the Bellman equation

2.1 $$Q(a,s)=R(a,s)+(1-\gamma_{a,s})\max_{\begin{array}{c} a^{\prime } \end{array}}Q(a^{\prime },T(a,s)).$$

The parameter $\gamma_{a,s}$ is interpreted as the rate of pruning of the search tree in a mean field approximation [26]. Correspondingly, rewards k steps ahead are discounted by a factor of ${\left(1-\gamma_{a,s}\right)}^{\left(1-k\right)}$. Scaling the state-action value Q by the inverse temperature β and applying a softmax function leads to the policy

2.2 $$\pi (a_{t}|s_{t})=\frac{e^{\beta Q(a_{t},s_{t})}}{\sum_{a^{\prime }}\;e^{\beta Q(a^{\prime },s_{t})}}.$$

Central to their work, Huys *et al.* defined a selective ‘Pruning’ version of this model to account for stronger pruning when participants encounter a large cost [26]. In our experiment, a large cost is defined as a reward of −100. In this model, which we will call the aversive pruning model, the $\gamma_{a,s}$ parameter takes two different values, a specific pruning rate $\gamma_{s}$ in the case of large costs and general pruning rate $\gamma_{g}$ in all other cases (2.3).

2.3 $$\gamma_{a,s}=\left\{ \begin{array}{ll} \gamma_{s}, & \text{if}\;R(a,s)=-100\\ \gamma_{g}, & \text{else} \end{array}\right.$$

### (c) Environment generation, selection and classification

Before the experiment, we created 800 environments, each one characterized by a directed network of six nodes and a starting node, with each edge of the network defining a possible move between two nodes. First, we created a pool of 60 000 strongly connected directed networks and uniformly sampled, for each link between two nodes, one of four possible rewards (−100,−20,20,140). Considering six possible starting nodes for each network, this yielded 360 000 environments. We then calculated for each environment a path maximizing the cumulative reward across eight consecutive moves. To reduce variation in the reward distributions, environments with a maximum reward in the upper and lower quartile were removed from the pool. To avoid trivial solutions (e.g. loops between two nodes), environments were rejected if the maximum path did not cover at least four distinct nodes. Finally, to exclude environments with myopic optimal solutions, we compared for each node on the optimal path, the reward of the optimal move with the reward of the alternative sub-optimal move. We required environments to have at least four moves in which the optimal move had the same or a lower direct reward then the sub-optimal one.

The final selection of environments was based on the sensitivity of the expected total reward to changes in the aversive pruning parameter. The aversive pruning sensitivity for each environment was examined by choosing a reference policy ((2.2), $\gamma_{g}=\gamma_{s}=0.35$ and β=0.03) and calculating the derivative of the expected reward with respect to the aversive pruning parameter $\gamma_{s}$. We then randomly selected 400 environments in the lowest and highest decentiles of aversive pruning sensitivity. We defined environments in the lowest decentile human-regretful as showing an aversive pruning bias in these environments leads to lower rewards. Environments in the highest decentile were called human-rewarding.

### (d) Matching the algorithmic performance

Rather than using an algorithm with super-human performance, we were interested in the effect of hybrid social learning between players that similarly discount future rewards and yet show different biases. We thus tuned the algorithm to have a comparable performance with a human player. In a pilot study, we estimated with a Bayesian model fit (see electronic supplementary material) the model parameter of a human player as $\gamma_{g}=0.20\;({\text{CI}}_{90}:(0.15,0.25))$, $\gamma_{s}=0.45({\text{CI}}_{90}:(0.32,0.58))$ and $\beta =0.012({\text{CI}}_{90}:(0.011,0.014))$. Note that $\gamma_{g}$ and $\gamma_{s}$ are comparable to the values reported by Huys *et al.* [26]. However, in our pilot data, we observed a lower inverse temperature β. One possible difference is that in Huys and colleagues’ work participants were extensively trained on one specific network, while in our work participants played in different environments (and each environment only once). Lower inverse temperature β in our study might then indicate more randomness in our participants' responses.

We designed a risk seeking algorithm with a bias inverse to humans but with comparable performance. We fixed $\gamma_{g}=0.5$ and $\gamma_{s}=0.05$ so as to have a comparable tree depth as human players, and then fitted β=0.0264 to match the performance of a human player on the pilot study. To mimic social learning, the algorithm used an additional heuristic at run time. First, a solution, i.e. a sequence of eight actions, was sampled using the parameters described above, then the total reward of this solution was compared with the one of the previous player. If the reward of the algorithm’s solution was greater or equal to the previous player’s reward, the algorithmic solution was played. Otherwise, an exact copy of the previous player’s solution was played by the algorithm.

### (e) Statistical analysis

We ran two separate hypothesis-driven regressions models, one on solution total reward (i.e. the sum of the rewards over the eight moves of a single round) and the other on whether a solution was optimal or not. Additionally, we ran exploratory regressions on the number of actions copied between solutions, modelled as Poisson distribution with a logarithmic link function. Different models were compared with a likelihood ratio test (*anova* function in R). We used a single model for both types of environments and consequently added interactions between each fixed effect of interest and the environment type. We used the human regretful environments as a baseline.

We ran a maximum-likelihood estimation of the pruning parameters (2.3) and inverse temperature (2.2) for each condition and environment type on 100 bootstrapped resamples of the solutions. We reported percentile confidence intervals and *p*-values based on the rate of samples satisfying the null hypotheses. 95% confidence intervals are reported throughout. The code of the statistical analysis and the corresponding data is published with this work.

### (f) Preregistration

Our preregistered hypotheses were that (H1) in human-only chains, individual solutions will improve across generations, within each environment, via social learning; (H2) we expected that placing the algorithm in the chain at generation two (GenHy2) will locally increase performance so that a score boost is observed in generation three (GenHy3) and following generations compared to the first generation (GenHy1); we expected our algorithmic manipulation (H3) to globally increase performance as measured by normalized score accrued in the game, (H4) to accelerate solution discovery as measured by the slope of score improvement and reduction of error compared to the global optimal solution, and (H5) to increase the likelihood of chains discovering the best solution. Furthermore, we expected the algorithmic intervention to not affect performance in human rewarding environments as people will judge that their solution is better than the algorithm’s (H6).

## 3. Results

### (a) Algorithm impacts the following generations, but the effect quickly decays

To investigate and compare the evolution of the performance of solutions in the different chains, we ran a linear mixed-effects model predicting the reward of a individual solution, by considering (a) the numeric position in the chain (generation), (b) individual generations following the algorithm and (c) the number of rounds participants had previously played (max 80) as additive effects. For the first two effects (a,b) we added an interaction with the environment type. Additionally we added random effects for the (d) individual participants and (e) individual environments. The round of a participant (c) was added to account for non-social learning of participants. We considered the first generation of the human regretful environments as the baseline. Algorithmic solutions were not considered in this analysis because they were part of our treatment.

We encoded the influence of the algorithm on the performance of following generations by adding two independent effects for the two generations directly following the algorithm (GenHy3 and GenHy4). All further generations were assigned a single effect (GenHy5+) and we considered solutions not following an algorithm in the chain as the baseline. This includes all solutions in human-only chains as well as the first generation (GenHy1) in the hybrid chains, where the algorithm has not yet been introduced. We selected this most parsimonious model (electronic supplementary material, table S1), because others that either included independent effects for all generations following the algorithm ($\chi^{2}=1.99$, df=2, p=0.37) or that included independent effects on the three generations (GenHy3, GenHy4 and GenHy5) following the algorithm ($\chi^{2}=5.13$, d.f.=6, p=0.52) did not significantly improve model fit.

**Figure 2.**
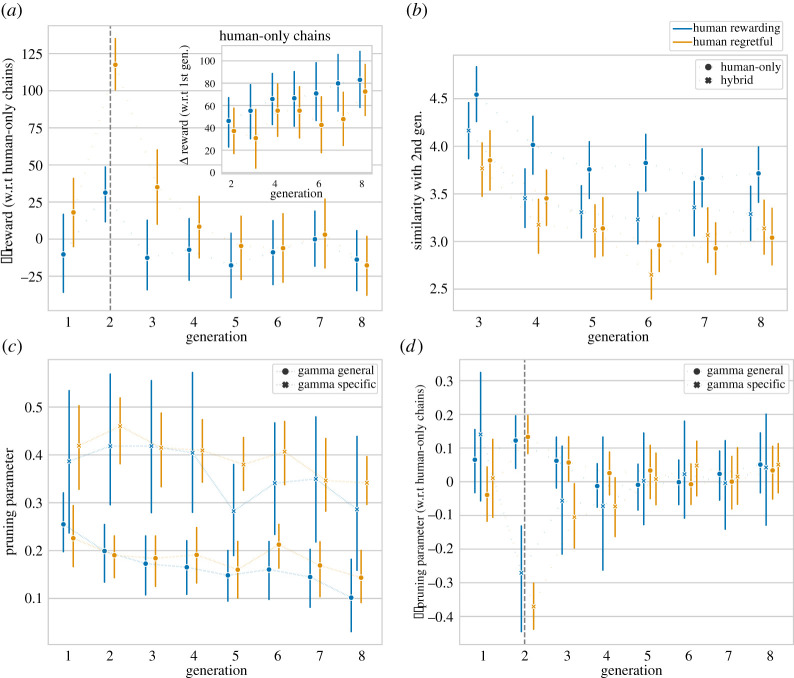
(*a*) Difference in performance between conditions (hybrid—human-only); (inset) performance improvement over generations within human-only chains in relation to the first generation; (*b*) average number of actions of solutions that match those of generation 2 within the same chain; (*c*) maximum-likelihood estimates of the pruning parameter for human-only chains; (*d*) difference of the maximum-likelihood estimates of the pruning parameter between conditions (hybrid—human-only). All panels share the same colour code. Vertical bars are indicating bootstrapped 95% confidence intervals. A dashed vertical line shows the algorithm’s position. (Online version in colour.)

As a first validation of our experimental set-up, we quantified the effect of social learning by investigating the impact of generation on reward. We found for human regretful environments an improvement of 3.867 (s.e.=1.244,t=3.109,p=0.002,CI=(1.429,6.305)) points from generation to generation, and for the human rewarding environment an additional improvement of 4.859 (s.e.=1.390,t=3.495,p<0.001,CI=(2.134,7.583)) points per generation. The inset in figure 2*a* depicts the average reward of solutions in human-only chains in relation to the reward of the first player in the chain. The positive slope indicates increase in performance over the eight generations suggesting the presence of social learning as predicted (H1) and the accumulation of higher performing solutions in later generations. Social learning appears to lead to larger increases in performance for ‘human rewarding’ environments where the human bias is beneficial.

Having found that social learning does occur, we investigated the impact of the algorithm on following generations in mixed chains. Figure 2*a* depicts the average within-environment reward difference between hybrid chains with human-only chains. We found for human regretful environments a significant effect (β(s.e.)=30.786(7.974),t=3.861,p<0.001,CI=(15.157,46.415)) for participants directly following the algorithm and a weak effect (β(s.e.)=13.225(7.922),t=1.669,p=0.095,CI=(−2.302,28.753)) for the second generation following the algorithm. No effect was found for the remaining generations (β(s.e.)=−2.473(5.094),t=−0.485,p=0.627,CI=(−12.457,7.511)). We did not find evidence for interactions of these effects with the environment type (see electronic supplementary material, table S2). Participants in the generation following the algorithm (generation 3) gained higher rewards than their counterparts in human-only chains. However, this effect appears to quickly wear off, suggesting a temporary boost in human performance due to hybrid social learning (H2). However, we found no evidence for global performance improvements in hybrid chains (H3) when considering the second half of the transmission chain (generation 5–8).

We then investigated how participants’ behaviour changed over the generations. We calculated a maximum-likelihood estimate of the aversive pruning model parameters, independently for each generation and each condition. To calculate confidence intervals around each point estimate, we bootstrapped 100 resamples. Figure 2*c* shows the pruning parameters estimates for human-only chains. For these chains, we ran a linear regression on the estimates and with generation as the only predictor, and found a significant reduction of general pruning rate $\gamma_{g}$ over generations for human rewarding (β=−0.017,p<0.01,CI=[−0.026,−0.0056]) and human regretful environments (β=−0.0078,p=0.05,CI=[−0.016,0.0013]), respectively. For the specific pruning rate $\gamma_{s}$, we found a similar reduction in both environments (human rewarding: (β=−0.017,p<0.01,CI=[−0.026,−0.0056]); human regretful (β=−0.0078,p=0.05,CI=[−0.016,0.0013])). These findings suggest that social learning led over the generations to solutions, which, on average, required more planning if done by individuals in isolation.

Given that the algorithm had a significant but temporary effect on following human performance, we investigated the effect of the algorithm on participants’ behaviour. Figure 2*d* shows the difference between parameter estimates in human-only and hybrid chains. Not surprisingly, we observed a difference between the algorithm and humans in generation two, as we designed the algorithmic parameter to show a different bias than participants. For solutions following the algorithm, we estimated for human regretful environments a lower specific pruning parameter (δ=−0.1,p=0.03,CI=[−0.2,−0.006]) and a higher general pruning parameter (δ=0.056,p=0.03,CI=[0.0014,0.13]). For human rewarding environments, we found in the same generation no significant differences between conditions (human rewarding: (δ=−0.046,p=0.24,CI=[−0.21,0.11]); human regretful: (δ=0.065,p=0.08,CI=[−0.019,0.13])). We found no significant difference between the two conditions in the following generations. On the one hand, these findings show that solutions directly following the algorithm are qualitatively different from the ones in human chains and that the algorithmic strategy is partially transmitted to participants following the algorithm. On the other hand, participants further down the transmission chain appear to reverse back to their typical strategies.

Finally, we investigated the rate at which participants in human-only and hybrid chains followed optimal strategies (see electronic supplementary material, figure S4). We ran a logistic regression with the same variables as previously described, on whether a solution was optimal. For hybrid chains, we found an increased rate at which optimal solutions are discovered in generation 3 (β(s.e.)=0.598(0.147),Z=4.059,p<0.001,CI=(0.310,0.887)) compared to human-only chains. However the difference quickly decayed and we did not find any significant difference in optimal solution discovery in final generations (β(s.e.)=0.598(0.147),Z=4.059,p<0.001,CI=(0.310,0.887)). Correspondingly, these findings do not support the hypotheses of a faster optimal solution discovery (H4) and sustained increase in discovery rate (H5) caused by the algorithm.

### (b) Algorithmic solutions are copied less, after controlling for scoring

Figure 2*b* depicts the average number of matching moves between second generation solutions (either human or algorithmic) and solutions in following generations (human). Despite their higher performance algorithmic solutions did not appear to be preserved, compared to their human counterparts. This finding may result from two opposite effects being at play. On the one hand, the higher reward of algorithm solutions could lead to a higher rate of copying. On the other hand, the mismatch with the inherent bias of participants might reduce copying. Note that algorithmic solutions can be either from the algorithm itself or the previous player if that solution was of higher performance. In chains with human regretful and human rewarding environments, the algorithm copied 21% and 43% of the solutions, respectively. This imbalance reflects the tuning of the algorithm towards human regretful environments.

To examine the mechanisms behind the algorithmic solution decay and a potential human content bias against algorithmic solutions, we conducted a set of exploratory analyses only on the third generation. We modelled the number of actions copied as a Poisson distribution with fixed effects for the previous solution’s (a) creator (algorithm or human) and (b) standardized reward (electronic supplementary material, table S2). We added random effects to account for covariation due to individual participants and environments. We found an increased rate of copying high scoring solutions (β(s.e.)=0.394(0.034),Z=11.423,p<0.001,CI=(0.326,0.461)) and a lower rate of copying algorithmic solutions (β(s.e.)=−0.189(0.041),Z=−4.625,p<0.001,CI=(−0.269,−0.109)). A model including an interaction between the two effects did not significantly improve model fit ($\chi^{2}=1.35$, df=2, p=0.51), suggesting that the two effects were additive. These findings suggest that once controlling for reward magnitude, algorithmic solutions were copied at a lower rate than human solutions. We found no significant interaction between the two main effects and type of environment.

We did not disclose to participants whether the previous solution they see is from an algorithm or another human, which ruled out any bias against algorithmic solutions beyond the characteristics of the solution itself. If this were the case, we should also expect to find reduced copying of those human solutions that happened to show a higher number of large costs (and an increased copying rate of high-performing solutions). We thus independently tested the hypotheses that both higher rewards and fewer large costs lead to increased copy rates. A model predicting the number of copied actions in generation 2 to 8 in human-only chains (electronic supplementary material, table S3) showed a positive effect of the previous solution’s reward (β(s.e.)=0.393(0.014),Z=28.162,p<0.001,CI=(0.366,0.420)) and a negative effect of the previous solution’s number of large costs (β(s.e.)=−0.040(0.011),Z=.3.544,p<0.001,CI=(−0.061,−0.018)). Both factors interacted with environment type, suggesting that they were stronger in human regretful environments.

These findings suggest a content bias in human social learning that favours higher rewards and fewer large costs. Consequently, solutions that do not match human bias, such as those of the algorithm, are less well preserved. In the next section, we explore whether reduced copying rates can be overcome by repeated exposure to algorithmic solutions.

### (c) An agent-based model: sustained performance improvements with repeated algorithmic exposure

**Figure 3.**
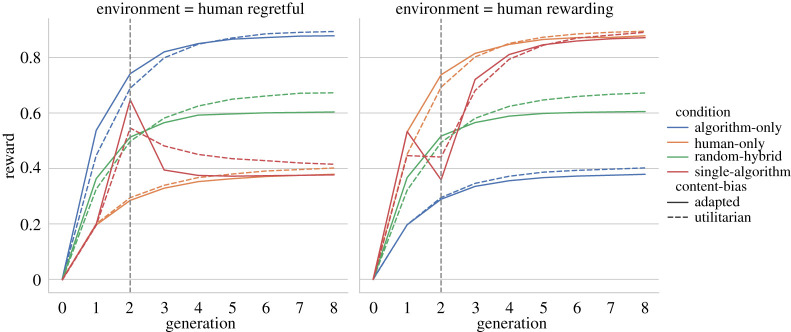
Average reward of the solutions of 100 000 modelled agents. Human-only chains are depicted in blue, algorithm-only in orange, hybrid chains with a single algorithm (as in the experiment) in green and randomly mixed hybrid chains in red. On the left panel the environment favours the algorithmic bias, on the right panel it favours the human bias. We compare two type of content bias, one with a bias for higher performing solutions (solid) and a second with an additional bias to match the specific bias of the agent. A dashed vertical line shows the algorithm’s position in the single-algorithm condition. (Online version in colour.)

We developed a simple agent-based model mimicking our experimental set-up to theoretically explore the impact of biases on social learning in hybrid cultural evolution (see electronic supplementary material, Methods for details). We modelled task solutions as points in a two-dimensional space with two independent qualities. The dimension $s^{g}$ represents the general quality of a solution, and the second dimension $s^{s}$ the specialization of a solution, i.e. how adaptive (or maladaptive) it is to a specific environment. Thus, human-like agents are adapted in ‘human-rewarding’ environments and algorithmic agents in ‘human-regretful’ environments. Notice that we modelled human and algorithmic agents symmetrically. Hence, ‘human-only’ chains on ‘human rewarding’ environments are symmetric in their performance with an ‘algorithmic-only’ on ‘human regretful’ environments and vice-versa (figure 3).

As in the experiment, we constructed chains of eight agents. Agents first assessed a perceived quality of the previous player's solution. Depending on this perceived quality, they decided to copy it or to sample an entirely new solution. The perceived quality determines the content bias of the agent. We compared two types of content biases. Agents with an ‘adapted’ content bias considered both the score of the previous solutions and the match with their specialization (dashed in figure 3). Agents with a ‘utilitarian’ content-bias considered only the score of the previous solution (solid in figure 3). Agents sampled new solutions from a distribution screed towards their adaptive bias. We initialized chains with a neutral solution.

Figure 3 shows the average reward over eight generations. Line colour represents chains composed of different agent types: human-only agents (blue), algorithmic-only agents (orange), and two hybrid chains with algorithmic and human agents. The ‘single-algorithm’ condition (green) represents the performance of hybrid chains similar to our experiment, with only one algorithm in generation 2. For ‘human-regretful’ environments (left panel), the algorithm in the second generation shows higher performance than their peers, and this boost is carried over to human-like agents in the next generation. Replicating our experimental results, the performance boost quickly decays back to the level of human-only chains (blue). The rate of decay is much faster for agents with an adapted content bias (solid green line) than for agents with a utilitarian content bias (dashed green line). For ‘human-rewarding’ environments (right panel), the introduction of the algorithm (green) leads to a performance drop compared to ‘human-only’ chains (blue). Again, performance converges in following generations.

The model allows us to investigate a condition that was not tested in our experiment, namely randomly mixing of humans and algorithms. Randomly mixed hybrid chains (red) show a performance in-between the performance of solely-adapted, e.g. algorithmic agents in regretful human environments, and solely-misadapted, e.g. human agents in human regretful environments. For these ‘random-hybrid’ chains, agents with a ‘utilitarian’ content-bias (red, dashed) converge to higher average performance than agents with an ‘adapted’ content-bias (red, solid). However, in the first two generations, agents in mixed chains with an ‘adapted’ content-bias have a slight edge over their peers.

The agent-based model shows that different chains converge to a fixed value irrespective of algorithmic participation at the beginning of the chain. Theoretically this can be easily shown by the underlying process being both a Markov chain and ergodic. We could reproduce the experimental finding that a performance boost of an adapted algorithm is not sustained by following humans—especially for human agents with a content bias against algorithmic solutions. However, our model suggests that, under specific conditions, improvement effects can be sustained in well-mixed hybrid social learning.

## 4. Discussion

In this work, we investigated the impact of algorithmic strategies on social learning using a transmission chain experiment. We adapted the decision-making task by Huys *et al.* [26] to a transmission chain paradigm to test whether introducing an algorithm to increase the diversity in decision strategies can improve collective performance via social learning. In this task, people are known to show an aversive pruning bias in exploring the decision tree. As expected, we found evidence of a performance improvement over generations due to social learning. Adding an algorithm with a different problem-solving bias than humans temporarily improved human performance but improvements were not sustained in following generations. While humans did copy solutions from the algorithm, they appeared to do so at a lower rate than they copied other humans’ solutions with comparable performance.

Our first contribution is expanding previous research in cultural evolution by suggesting a relatively unexplored area of investigation, namely hybrid social learning. Scholars of cultural evolution have long investigated how social learning could lead to the unmatched explosion of human cultural complexity in comparison to non-human animals [32,45,46]. Similarly, we might ask if the advent of self-learning algorithms can influence cultural trajectories via hybrid human–algorithm social learning. Going one step beyond prior work that looked into cultural evolution via digital technology [8,9], we suggest that in a hybrid society, algorithms may not be just a medium for cultural transmission, but may play an active role in the production of new cultural artefacts. In particular, we suggest that successful hybrid social learning may occur when algorithms, either by design or by self-learning, show different biases than their human counterparts. Although many algorithms quickly adopt human biases [47–49], several others can increasingly learn from direct interactions with the environments rather than from human data, thereby potentially showing new behaviours and biases. Greater variance in problem-solving and copying skills has been associated with greater cultural variance [50,51] and—as long as there are selection biases with regard to who to copy from—greater innovation. We looked at particular situations where human biases are known to constrain human performance [26], and therefore humans could most benefit from observing an algorithmic strategy.

In our experiment, we tested these hypotheses by introducing algorithmic players that adopted different decision-making strategies than human players. Investigating hybrid groups of human and algorithmic players provides the experimenter with the advantage of closely controlling the behaviour of algorithmic agents while observing the effect on the rest of the group [16,52,53], yet, to the best of our knowledge, bots have not prominently featured in transmission chain experiments.

Our second contribution lies in our empirical findings. We showed that participants did not preserve algorithmic solutions if they were incongruent with their bias in avoiding large costs. Although human and algorithmic biases have been thoroughly investigated in their respective fields (psychology/economics and computer science), how the two interact is still poorly understood. We show that learning from algorithms might be limited by the specific task and cognitive biases characterizing human players. In our experiment, higher-performing solutions that were incongruent with human biases showed lower copying rates, and were consequently lost over generations. Such a preference for copying congruent solutions may limit the accumulation of algorithmic solutions into human repertoires [45,51]. This result is in agreement with a well-replicated finding in transmission chain experiments. Many cultural traits, such as drawings [38], stories [39], norms [54,55] and language [56], converge over generations independently from the initial conditions of the chain. For example, the effect of implausible values provided by human confederates in an estimation task has been shown to quickly dissipate [55]. What these studies show is that in the absence of a difference in fitness of the cultural artefacts, the equilibrium distribution of a transmission chain directly matches the human bias [57]. However, these previous studies did not control for the solution’s fitness (i.e. accuracy or informativeness). In language, for example, trade-offs of informativeness and compressibility determine linguistic structure [56] and likewise human biases and external fitness can be in disagreement [58].

Our novel contribution to this previous work is using an algorithm that provides a solution that mismatches human biases but that is at the same time highly accurate in the task environment. Analytical work of Griffiths *et al.* suggests that, in agreement with our findings, when participant bias and solution fitness go against each other, superior solutions will not be maintained in conditions of moderate to high transmission noise [59]. In a follow-up work, Thompson and Griffiths modelled cultural evolution in transmission chain experiments as being influenced by attraction towards preexisting biases and local innovations [58]. The authors experimentally showed that, if the two are in conflict, participants’ solutions converge to a middle ground. While their work models the effect of inductive biases on artefacts, our work focuses on the effect of content biases on copying. Yet, biases hinder the discovery of optimal solutions in both cases. Our work goes beyond their findings and suggests that even if an algorithm aids humans in achieving optimal solutions, human bias in what to copy can lead to those solutions being quickly lost in successive human–human transmissions, unless repeated exposure takes place. It is important to note that while human bias sped up the dissipation, convergence itself is guaranteed in transmission chain experiments with a bounded solutions space and with a non-zero chance of cultural loss.

We suggest that hybrid social interaction among human and algorithmic players may play an increasingly critical role in today’s digital society. Such human–algorithmic interaction can have diverse modes, such as observation [60], conversation [61] or even teaching [62]. Previous research on cultural evolution using transmission chains has found that the accumulation of artefacts of increasing performance can accrue through all of those modes [63]. Accordingly, we focus on observational learning as the most simple form of cultural transmission. Investigating the effect of algorithms on human behaviour in the wild [11] has the obvious advantage of validity but renders investigating causalities challenging. In this work, we follow a tradition in cultural evolution that tries to generalize laboratory findings to the real world [35,64]. Although the limitations of such extrapolations are known [65], investigating human–algorithmic social learning in the laboratory is the first step to study how these phenomena might unfold in the real world, and how interactions in hybrid social systems may foster or hinder innovations and collective performance.

Designing algorithms to nudge collective behaviour may add to an already long list of ethical concerns in AI [47,49,66,67]. Our results further suggest that even algorithms that could objectively improve human performance might be limited. Content and context biases (i.e. what people are more likely to copy and who they are willing to copy from) might limit hybrid social learning, especially in uncertainty, high cognitive demand, or high time pressure (i.e. high transmission noise [58]). Under these conditions, humans are more likely to follow well-known and adaptive biases [18,68]. This by no means suggests that algorithms do not have an impact on human cultural evolution. Many humans routinely and repeatedly interact with AI systems that operate on a global scale. Indirect influence on human behaviour has been low in this work, i.e. there were diminishing effects on humans who interacted with humans who interacted with an algorithm. However, even minor effects might have pronounced consequences in an interconnected human–algorithmic hybrid society. More research is needed to investigate the diffusion of algorithmic behaviour and artefacts into human culture.

Importantly, we acknowledge the limitations of our study, both in terms of generalizability and sample size. Future studies will need to address whether AI-human collaboration may be more successful in other domains or simpler tasks. In our experiment, we were interested in isolating cultural transmission by exposing participants to one previous solution only. This may limit the generalizability of our study. Outside the laboratory, people can copy from multiple models, which may give them the option to compare alternative solutions. Also, while in our experiment we tested the effect of a single algorithmic player, the frequency of encountering algorithmic generated solutions in the real world may be higher. For instance, in the case of Go, it is known that professional players include algorithms in their daily training. Our agent-based model (figure 3) predicts that sustained improvement in performance might be observed with greater chances to copy from algorithms, although more work is needed. Finally, in our experiment people visited each environment only once. This likely reduced the effect of individual learning as well as giving participants inadequate feedback on their performance. Repeated unsuccessful feedback with the same environment before being exposed to an algorithmic solution might give participants additional opportunities to copy the algorithm, when algorithmic solutions are valuable.

In this work, we focused on the transmission of behaviour, rather than the transmission of strategy itself. Social learning seems to be more effective when copying exact behaviours rather than reasoning and decision strategies [32,69,70]. Understanding why a solution works is not a prerequisite for successful cultural evolution [34]. Yet, more explicit communication between model and observer—e.g. in the case of teaching [7]—could allow for better transmission of strategy. For example, communication of intention can improve human–algorithm cooperation [71]. Correspondingly, we speculate that an algorithm that communicates the reasoning behind a solution might be copied at a higher rate and allow following humans to better critically appraise their preexisting beliefs. Professional Go software allows players to play out different moves, comparing the consequences of an apparent alien move with a more traditional strategy. Nevertheless, in Go and chess, humans might still be limited and influenced by their biases in learning new strategies from an algorithm. An exciting potential could lie in algorithms that combine human-like [72] and alien play in order to improve the learnability of algorithmic solutions.

To conclude, in this work, we found limited influence of bots on human cultural evolution. Our findings do not exclude the possibility of algorithmic influences on human culture, but draw some limiting conditions. The relationship between biased human strategies and algorithmic strategies derived by self-play might look different outside the laboratory where more complex AI algorithms are at play. However, studying these phenomena in controlled environment is an important first step to understanding hybrid social learning. In this study, we suggested that differences between human and algorithmic behaviour might be relevant for the emerging properties of cultural evolution.

## Data Availability

All scripts used in this study are openly accessible through https://github.com/StochasticBiology/boolean-efflux.git. Raw data, processed data, preregistration and code for the analysis is available on OSF: https://osf.io/5j6es/. The data are provided in electronic supplementary material [73].
